# Immune checkpoint inhibitors: recent progress and potential biomarkers

**DOI:** 10.1038/s12276-018-0191-1

**Published:** 2018-12-13

**Authors:** Pramod Darvin, Salman M. Toor, Varun Sasidharan Nair, Eyad Elkord

**Affiliations:** 1 Cancer Research Center, Qatar Biomedical Research Institute, College of Science and Engineering, Hamad Bin Khalifa University, Qatar Foundation, Doha, Qatar; 2 Institute of Cancer Sciences, University of Manchester, Manchester, UK

## Abstract

Cancer growth and progression are associated with immune suppression. Cancer cells have the ability to activate different immune checkpoint pathways that harbor immunosuppressive functions. Monoclonal antibodies that target immune checkpoints provided an immense breakthrough in cancer therapeutics. Among the immune checkpoint inhibitors, PD-1/PD-L1 and CTLA-4 inhibitors showed promising therapeutic outcomes, and some have been approved for certain cancer treatments, while others are under clinical trials. Recent reports have shown that patients with various malignancies benefit from immune checkpoint inhibitor treatment. However, mainstream initiation of immune checkpoint therapy to treat cancers is obstructed by the low response rate and immune-related adverse events in some cancer patients. This has given rise to the need for developing sets of biomarkers that predict the response to immune checkpoint blockade and immune-related adverse events. In this review, we discuss different predictive biomarkers for anti-PD-1/PD-L1 and anti-CTLA-4 inhibitors, including immune cells, PD-L1 overexpression, neoantigens, and genetic and epigenetic signatures. Potential approaches for further developing highly reliable predictive biomarkers should facilitate patient selection for and decision-making related to immune checkpoint inhibitor-based therapies.

## Cancer immunotherapy: Markers of success

Biomarkers that predict a patient’s response to cancer immunotherapy are being developed, and may allow individual tailoring of therapies. Cancer cells can suppress the immune system by activating immune checkpoints, signals that stop the immune system from attacking the host. Immunotherapy, a recently developed treatment, inactivates immune checkpoints, restoring the patient’s immune response against the tumor. Although promising, immunotherapy has a low response rate, and sometimes triggers severe auto-immune reactions. Eyad Elkord at Qatar Biomedical Research Institute in Qatar and coworkers have reviewed biomarkers that could be used to predict individual effects of immunotherapy, identifying nonresponders and preventing adverse immune reactions. They discuss markers based on factors such as immune response level and individual tumor genetics, among others. With further research and testing, these biomarkers could improve the efficacy of this promising new cancer therapy.

## Introduction

The development of immune checkpoint inhibitors (ICIs) is a revolutionary milestone in the field of immuno-oncology. Tumor cells evade immunosurveillance and progress through different mechanisms, including activation of immune checkpoint pathways that suppress antitumor immune responses. ICIs reinvigorate antitumor immune responses by interrupting co-inhibitory signaling pathways and promote immune-mediated elimination of tumor cells.

Ipilimumab, which targets cytotoxic T-lymphocyte antigen-4 (CTLA-4), was the first approved immune checkpoint inhibitor for treating patients with advanced melanoma^1–3^. This antibody prevents T-cell inhibition and promotes the activation and proliferation of effector T cells. Following the approval of ipilimumab, other antibodies that target immune checkpoints were examined. Currently, hundreds of phase I and II clinical trials and phase III/IV clinical trials are being carried out across the globe to evaluate the efficacy of multiple ICIs as monotherapy or in combination (details of phase III/IV trials are given in Table 1).

**Table 1:** Immune checkpoint inhibitors in phase III and IV clinical trials

Sl No	Drug	Cancer type	Clinical trial ID
1	Pembrolizumab (Anti-PD-1)	NSCLC	NCT03134456, NCT02220894, NCT02142738, NCT02864394, NCT03302234, NCT01905657, NCT02504372, NCT02775435, NCT02578680
2		Small cell lung cancer	NCT03066778
3		Head and neck squamous cell carcinoma	NCT02252042, NCT03040999, NCT02358031
4		Renal cell carcinoma	NCT03142334, NCT02853331
5		Gastric adenocarcinoma	NCT02370498
6		Nasopharyngeal neoplasms	NCT02611960
7		Urothelial carcinoma	NCT02853305, NCT03244384, NCT02256436, NCT03374488, NCT03361865
8		Colorectal cancer	NCT02563002
9		Pleural mesothelioma	NCT02991482
10		TNBC	NCT02819518, NCT03036488, NCT02555657
11		Esophageal neoplasms	NCT03189719, NCT02564263
12		Multiple myeloma	NCT02579863, NCT02576977
13		Gastric and gastroesophageal junction cancer	NCT03019588, NCT03221426
14		Gastric adenocarcinoma	NCT02494583
15		Melanoma	NCT02362594, NCT01866319
16		Hodgkin lymphoma	NCT02684292
17		Hepatocellular carcinoma	NCT02702401, NCT03062358
18		Lung cancer	NCT03322540
19		Head and neck cancer	NCT03358472
20	Nivolumab (Anti-PD-1)	NSCLC	NCT02041533, NCT01642004, NCT01673867
21		Mesothelioma	NCT03063450
22		Non-Hodgkin lymphoma	NCT03366272
23		Metastatic clear cell renal carcinoma	NCT01668784
24		Head and neck cancer	NCT02741570, NCT03342352
25		Lung cancer	NCT03348904
26		Melanoma	NCT03068455, NCT01844505
27	Ipilimumab (Anti-CTLA-4)	NSCLC	NCT03469960, NCT03351361, NCT02785952, NCT03302234
28		Squamous cell lung carcinoma	NCT02785952
29		Mesothelioma	NCT02899299
30		Gastric cancer Gastroesophageal junction cancer	NCT02872116
31		Metastatic melanoma	NCT03445533, NCT00636168, NCT01274338, NCT02339571, NCT02506153, NCT02224781, NCT00094653
32		Metastatic non-cutaneous melanoma	NCT02506153
33	Avelumab (Anti-PD-L1)	NSCLC	NCT02576574, NCT02395172
35		Urothelial cancer	NCT02603432
35		Diffuse Large B-cell lymphoma	NCT02951156
36		Renal cell cancer	NCT02684006
37		Gastric and gastroesophageal junction cancer	NCT02625623, NCT02625610
40	Atezolizumab (Anti-PD-L1)	Ovarian cancer, fallopian tube cancer Peritoneal neoplasms	NCT03038100, NCT02839707, NCT02891824
41		NSCLC	NCT02813785, NCT02008227, NCT02367781, NCT02366143, NCT02409342, NCT02486718, NCT02367794, NCT03191786, NCT02409355, NCT02657434, NCT03456063
42		Extensive stage small cell lung cancer	NCT02763579
43		TNBC	NCT03197935, NCT02425891, NCT03125902, NCT03281954
44		Renal cell carcinoma	NCT02420821, NCT03024996
45		Bladder cancer	NCT02302807
46		Squamous cell carcinoma of the head and neck	NCT03452137
47		Urothelial carcinoma	NCT02807636
48		Transitional cell carcinoma	NCT02450331
49		Prostatic neoplasms	NCT03016312
50	Durvalumab (Anti-PD-L1)	NSCLC	NCT02352948, NCT03003962, NCT02453282, NCT02273375, NCT02542293, NCT03164616, NCT02125461,
51		Squamous cell lung carcinoma	NCT02154490, NCT02551159
52		Recurrent or metastatic PD-L1 positive or negative SCCHN	NCT02369874
53		Recurrent squamous cell lung caner	NCT02766335, NCT02154490
54		Urothelial cancer	NCT02516241
55		Advanced solid malignancies	NCT03084471
56		SCCHN, hypo pharyngeal squamous cell carcinoma, laryngeal squamous cell carcinoma	NCT02551159, NCT03258554
57	REGN2810 (Anti-PD-1)	NSCLC	NCT03409614, NCT03088540
58	BMS-936558 (Anti-PD-1)	Unresectable or metastatic melanoma	NCT01721746, NCT01721772
59	SHR1210 (Anti-PD-1)	NSCLC	NCT03134872
60		Nasopharyngeal neoplasms	NCT03427827
61	KN035 (Anti-PD-L1)	Biliary tract neoplasms	NCT03478488
62	IBI308 (Anti-PD-1)	Squamous cell lung carcinoma	NCT03150875
63	PDR001 (Anti-PD-1)	Melanoma	NCT02967692
64	Anti-PD-1	Metastatic melanoma	NCT02821013
65	BGB-A317 (Anti-PD-1)	NSCLC	NCT03358875
66		Esophageal squamous cell carcinoma	NCT03430843
67		Hepatocellular carcinoma	NCT03412773
68	BCD-100 (Anti-PD-1)	NSCLC	NCT03288870
70	JS001 (Anti-PD-1)	Metastatic melanoma	NCT03430297

Pembrolizumab and nivolumab, ICIs that target programmed death-1 (PD-1), showed promising results in melanoma and non-small cell lung carcinoma (NSCLC) patients, with an objective response rate (ORR) of 40–45%^4–6^. Additionally, urothelial bladder cancer patients treated with PD-1/PD-L1 inhibitors showed an increase in overall response rate, between 13 and 24%^7^. In triple-negative breast cancer (TNBC) patients, the response to PD-1 inhibitors was relatively moderate (19%)^8^. In contrast, in relapsed or refractory Hodgkin’s lymphoma, nivolumab showed an objective response rate of 87% with 17% complete response^9^. Pembrolizumab and nivolumab are currently under phase IV clinical trials for treating various malignancies (Table 1).

Despite the success of anti-CTLA-4 and anti-PD-1/PD-L1 therapies, only a fraction of patients benefit from ICIs. Antitumor immunity, regulated through complex factors in the tumor microenvironment (TME), could create variable immune responses. The TME is segregated into three major types based on the infiltration of immune cells: immune desert, immune excluded and immune inflamed^10^. These phenotypes have their own mechanisms for preventing immune responses from eradicating tumor cells^10^. Immune deserts are characterized by the absence of T cells in the TME and the lack of suitable T cell priming or activation. The immune excluded phenotype exhibits the presence of multiple chemokines, vascular factors or mediators and stromal-based inhibition; however, accumulated T cells are unable to infiltrate the TME. Immune inflamed tumors demonstrate infiltration of multiple immune cell subtypes^10^.

Accumulating evidence suggests that only a fraction of cancer patients benefit from checkpoint inhibitors, and severe immune-related adverse events (irAEs) are seen in some patients undergoing ICI therapy^11^. irAEs are due to the inhibition of immune checkpoints that reinforce the normal physiological barriers against autoimmunity, leading to various local and systemic autoimmune responses. Therefore, the development of predictive biomarkers is critical for differentiating responders and nonresponders to avoid any adverse effects. Ongoing clinical studies are aiming to develop predictive biomarkers for better treatment outcomes and less irAEs.

Predictive biomarkers could determine the outcome of therapy in a patient before the initiation of a proposed therapy. These biomarkers should indicate whether a patient would benefit from a particular checkpoint monotherapy or if there is a need for combination therapy. In this review, we discuss biomarkers that predict the response to various ICI therapies in cancer.

## Immune cells

Immune inflamed tumors have a high degree of response to immunotherapy. Reports suggest that immune inflamed tissues are more sensitive because ICIs can activate immune reactions and inhibit immune evasion/suppression. Studies confirmed that the response to ICI therapy is related to tumor-infiltrating lymphocytes (TILs) and other immune cells in the TME^12^. 

Analyses of peripheral blood is a noninvasive method with good potential to predict treatment outcomes after immune therapies. Reports have shown that in various malignancies, increased tumor-infiltrating immune cells and peripheral blood absolute lymphocyte count (ALC) can be utilized as predictive biomarkers^13–15^. The role of ALC as a predictive biomarker has been validated in metastatic melanoma patients treated with ipilimumab. Patients with 1.35-fold higher ALC values from baseline in the first 2 weeks of treatment had significantly higher overall survival^16^. In ipilimumab-treated patients, overall progression-free survival was associated with a low serum lactate dehydrogenase value (LDH ≤ 1.2-fold), a low absolute monocyte count (AMC < 650 cells/µL), a low myeloid-derived suppressor cell count (MDSCs < 5.1%), a high absolute eosinophil count (eosinophils ≥ 50 cells/µL), a relative lymphocyte count < 10.5% and baseline CD4^+^CD25^+^FOXP3^+^ Tregs ≥ 1.5% in the peripheral blood^15,17^. Multiple studies validating the applicability of LDH as a predictive biomarker showed that patients with elevated levels of LDH also responded to ICIs^18^. Studies have reported that LDH can be used as a potential predictive biomarker for overall survival but not as a prognostic biomarker^15,16,19^. CyTOF-based immune profiling of peripheral blood samples collected from anti-CTLA-4 and anti-PD-1-treated melanoma patients showed a distinct set of biomarkers in response to therapy^20^. This study suggested that the abundance of CD4^+^ and CD8^+^ memory T cells was a predictive biomarker for anti-CTLA-4 therapy and the abundance of CD69^+^ and MIP1β^+^ NK cells was a predictive biomarker for anti-PD-1 therapy^20^. CyTOF analyses of anti-PD-1-treated melanoma patients showed an involvement of CD14^+^CD16^−^HLA-DR^hi^ cells in therapy response and progression-free survival (PFS)^21^. An increase in circulating CD4^+^, CD8^+^ T cells and ALC, 2 to 8 weeks after treatment initiation with ipilimumab, was reported in melanoma patients with better clinical outcomes^22^. Apart from the circulating CD8^+^ T cells, CD8^+^ effector memory type-1 T cells were also reported as predictive biomarkers for ipilimumab-treated stage IV melanoma patients^23,24^.

**Fig. 1:**
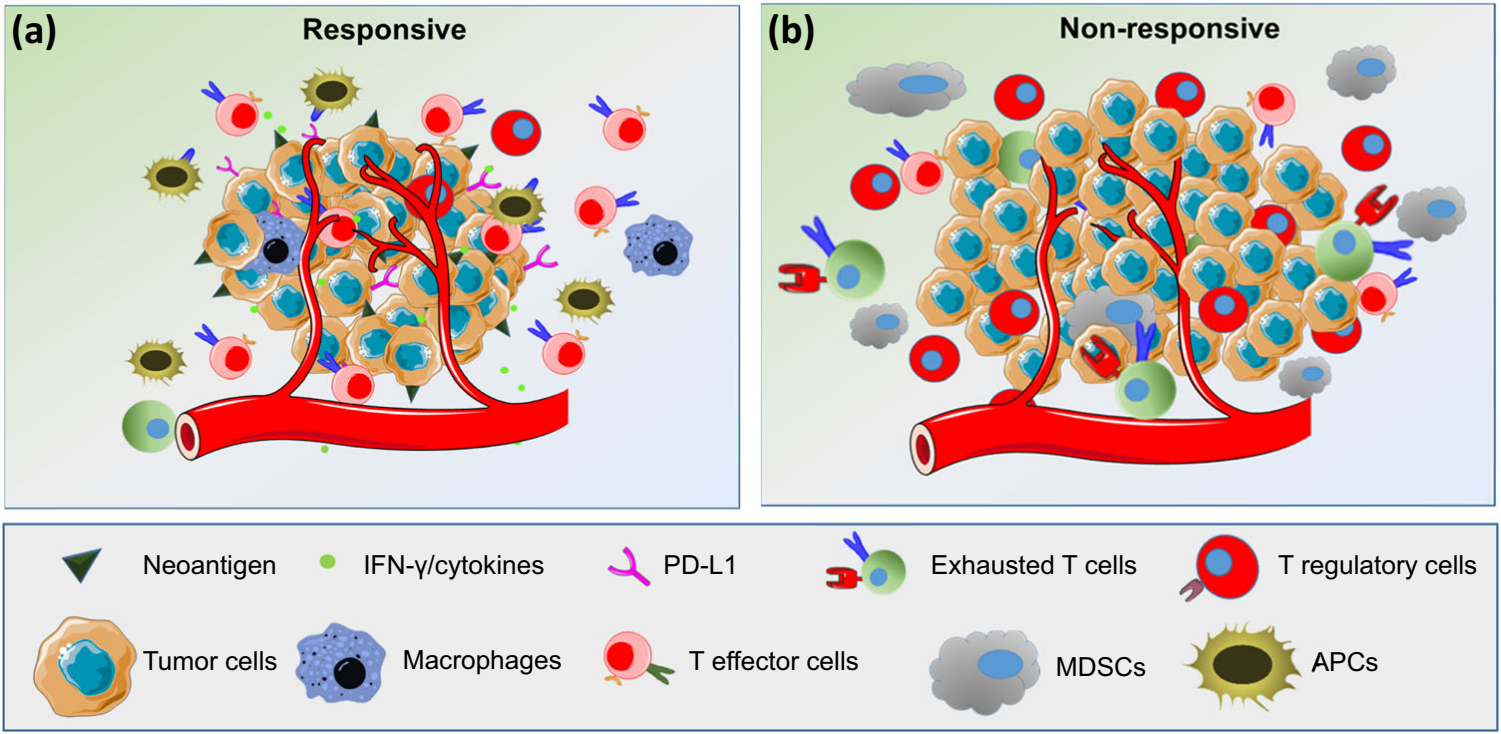
Overview of predictive biomarkers for response to ICIs. The response to immune checkpoint inhibitors varies depending on the TME. In the responders, tumors have a high neoantigen load, high levels of TILs, especially effector cells, a high Teff to Treg ratio, low MDSC levels and increased secretion of IFN-γ and other cytokines (**a**). In nonresponders, the TME contains high levels of immunosuppressive cells, such as Tregs and MDSCs, and very low levels of NK cells and activated lymphocytes (**b**)

The presence of TILs in various malignancies can be used as potent predictive biomarkers for response to ICIs^13,14^. Tumors with increased TILs are a major hallmark of the immune inflamed phenotype, and they exhibit improved immune-mediated elimination of tumor cells. In ipilimumab-treated melanoma patients, TILs were significantly increased from baseline in a therapy-responsive group, confirming their significance in response to ICIs^25^. To explain the role of immune cells in the treatment response, a study was carried out using 52 lymph nodes and 34 cutaneous/subcutaneous metastatic surgical samples collected from 30 metastatic melanoma patients receiving ipilimumab^26^. In this study, Balatoni et al.^26^ examined 11 immune cell subsets in the TME and their post-therapy responses. Interestingly, 7 out of 11 immune subsets positively correlated with an increase in the overall survival rate. These subsets included CD4^+^ T cells, CD8^+^ T cells, CD20^+^ B cells, cells expressing CD134^+^ and CD137^+^ activation markers, FOXP3^+^ T cells and NKp46^+^ cells. Notably, subcutaneous and cutaneous metastatic tissues, compared to lymph nodes, showed distinct immune cell infiltration. In subcutaneous and cutaneous samples, the presence of CD16^+^ and CD68^+^ cells positively correlated with therapy response as well as prolonged survival. In contrast, in the lymph nodes, CD45RO^+^, PD-1^+^, CD16^+^, and CD68^+^ cells correlated only with increased survival^26^. In addition to the abundance of FOXP3^+^ Tregs, the ratio of effector T cells (Teffs) to Tregs is reported to be a more specific predictive biomarker for anti-CTLA-4 immune therapies^27,28^. Immune profiling of TILs using multiparametric flow cytometry in metastatic melanoma patients showed that PD-1^hi^CTLA-4^hi^ in CD8^+^ T cells predict the response to anti-PD-1 therapy^29^. This study is supported through the identification of both transcriptionally and functionally distinct CD8^+^PD-1^+^ T-cell subpopulations in NSCLC patients, showing predictive potential for anti-PD-1 therapy^30^. Additionally, intratumoral and peripheral CD4^+^FOXP3^−^PD-1^hi^ nonconventional Tregs in NSCLC as well as melanoma patients were reported as prognostic biomarkers for anti-PD-1 and anti-CTLA-4 therapies^31^. Anti-CTLA-4 therapy induced an immune inflamed phenotype via expansion of intratumoral and systemic CD4^+^FOXP3^−^PD-1^hi^ Tregs that were reduced with anti-PD-1 therapy and improved the overall antitumor response^31^. Moreover, PD-L1^+^CD4^+^CD25^+^ Tregs predict responses to PD-1/PD-L1 blockade in NSCLC patients^32^. Figure 1 shows an overview of how the presence of various immune cell subsets in the TME may contribute to the differential responses to ICIs in responders and nonresponders.

Pembrolizumab in advanced melanoma patients showed that pre-existing CD8^+^ T cells in the TME are required for better tumor regression^33^. The presence of an immune excluded phenotype with an abundance of immune cells at invasive margins or stroma is also associated with clinical benefits. The spatiotemporal dynamics of CD8^+^ T cells are also an important factor for better treatment outcomes. Analysis of pretreatment samples collected from patients undergoing PD-1/PD-L1 therapy showed a relatively higher abundance of CD8^+^ T cells at the invasive margins in therapy responders. These pretreatment samples show an immune excluded phenotype through increased accumulation of T cells on the invasive margin without effective infiltration. Moreover, serially sampled tumors during therapy showed an increase in CD8^+^ T cells at the invasive margin and then in parenchyma in the response group^33^. This increase in CD8^+^ T cells may be due to the negative regulation of PD-1/PD-L1 by ICIs, which resulted in either the infiltration of immune cells or the enhanced proliferation of CD8^+^ T cells^33^. Additionally, it has been reported that in lung cancer patients, CD3^+^, CD4^+^ and CD8^+^ T-cell infiltration to deep tissues significantly correlated with longer overall survival^34^. Metastatic breast cancer patients treated with atezolizumab showed an increased ORR related to stromal TILs^35^. The predictive potentials of stromal TILs were confirmed in the KEYNOTE-086 study; significantly higher levels of stromal TILs were associated with the anti-PD-1 therapy response in metastatic triple-negative breast cancer patients^36^.

**Fig. 2:**
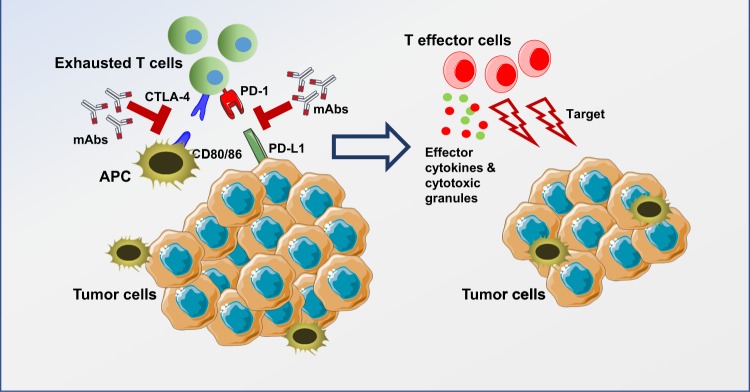
Immune checkpoint blockade for T-cell activation. Immune checkpoints, including PD-1 and CTLA-4, expressed on activated T cells lead to inhibition of T-cell activation upon binding to their ligands on tumor cells/antigen-presenting cells. These interactions can be blocked using monoclonal antibodies, leading to the activation of T cells targeting tumor cells through the release of effector cytokines and cytotoxic granules.

CTLA-4 blockade activates T cells to target malignant cells. CTLA-4 is constitutively expressed in T cells and attenuates immune responses when bound to CD80 or CD86 on the surface of antigen-presenting cells (APCs) (Fig. 2). Analyses of pre- and post-treated surgical tissues and peripheral blood showed that the inducible costimulator (ICOS) pathway is activated upon anti-CTLA-4 therapy^37^. This overexpression of ICOS (CD28/CTLA-4 Ig superfamily) resulted in an increase in ICOS^+^ T cells in both tumor and blood samples^37^. In tremelimumab-treated breast cancer patients, increased CD4^+^ICOS^+^ and CD8^+^ICOS^+^ T cells were observed in peripheral blood^37^. The ratio of FOXP3^+^ Treg cells to ICOS^+^ T cells was also increased in therapy-responsive patients^37^. Moreover, in patients exhibiting clinical benefits, there was an increase in the frequency of CD4^+^ICOS^+^Teff cells. These cells express T-bet and produce IFN-γ, strengthening immune responses in anti-CTLA-4 therapy^38–40^.

## PD-L1 overexpression

Interactions between PD-1 and its ligands, B7-H1/PD-L1 and B7-DC/PD-L2, lead to T-cell inactivation to maintain immune homeostasis and prevent autoimmunity. PD-1/PD-L1 pathway activation is related to the immune inflamed phenotype^41^. IC ligands are commonly found on tumor cells, and these interactions work in tandem with elevated tumor infiltration of immunosuppressive cells to support tumor escape from active T-cell responses^42^. Therefore, blocking the PD-1/PD-L1 inhibitory pathway can activate T cells in the TME, releasing inflammatory cytokines and cytotoxic granules to eliminate tumor cells (Fig. 2).

The direct approach to check responsiveness to PD-1/PD-L1 therapy in patients is to detect the expression levels of PD-L1 in tumor tissues. Teng et al.^43^ proposed four different classifications of TME based on the presence of TILs and PD-L1 expression. They classified PD-L1-positive tumors with TILs as a type I tumor microenvironment and proposed it to be most likely to respond to immune checkpoint blockade.

IHC analyses performed on patients with metastatic melanoma, NSCLC, colon cancer, renal cell carcinoma and prostate cancer who underwent PD-1/PD-L1 targeting therapy suggested PD-L1 overexpression as a potential biomarker. An open-label Phase II clinical trial of pembrolizumab in NSCLC reported that progression-free survival and overall survival were higher in patients with PD-L1 expression in at least 50% of tumor cells^44^. Notably, elevated levels of PD-L1 expression in the TME do not correlate with worse differentiation and poor prognosis. High PD-L1 expression is often accompanied by IFN-γ-secreting TILs in some cancers^45^. However, Aguiar et al. suggested that PD-L1 overexpression may not be a robust biomarker for the response to ICIs in all cancers, as PD-L1-negative tumors can also respond to mAbs targeting PD-1/PD-L1 interactions. Therefore, to date, PD-L1 overexpression as a prerequisite for initiation of PD-1/PD-L1 checkpoint blockade is not established as a potent biomarker for determining responsiveness to anti-PD-1/PD-L1 based immunotherapy.

Investigating PD-L1 expression has some limitations that need to be considered. PD-L1 expression is known to be both spatial and temporal, and it is also expressed on other immune cells, including antigen-presenting cells. One plausible approach to counter these limitations is to perform PD-L1 expression analyses on circulating tumor cells (CTCs) in peripheral blood samples from cancer patients. Interestingly, PD-L1^+^ CTCs were found to be higher than PD-L1^+^ cells in the TME of NSCLC patients (83% vs. 41%), and no correlation was observed between tissue and CTC PD-L1 expression^46^. Therefore, further investigations are warranted to establish PD-L1 expression on CTCs as a predictive biomarker.

## Neoantigens

Acquired mutations during cancer progression have promise in detecting efficiency of and resistance to therapy. Mutations in the protein-coding regions of DNA generate truncated proteins termed ‘‘neoantigens.’’ Neoantigens result in a higher degree of foreignness to cells, which helps immune cells readily target and eliminate tumor cells. Various neoantigens that confer therapy efficacy could be potential biomarkers for predicting the clinical activity of ICIs. A retrospective study on stage I/II and stage III/IV lung cancer samples showed that high neoantigen burden is associated with the longest overall survival (*P* = 0.025)^47^. Moreover, intratumoral heterogeneity analyses showed that high neoantigen-expressing clones were homogenous with the highest differential expression of PD-L1 and IL-6^47^. Additionally, CD8α and β, STAT1, TAP-1 and 2, CXCL-10, CXCL-9, granzyme–B, –H, and –A, and IFN-γ were upregulated in the high neoantigen-expressing clones^47^. Overexpression of IFN-γ, IDO, and Th1-associated markers was reported in ipilimumab-treated patients with favorable clinical outcomes. Resistance to CTLA-4 therapy was observed with a loss in IFN-γ signaling in CD8^+^ T cells. These findings confirm that the immune-mediated elimination of tumor cells could be proportional to the neoantigen load. Neoantigens exhibiting high-affinity binding with MHC and TCR are highly eliminated neoantigens^48^. Moreover, acquired resistance to ICI can also be predicted through neoantigen landscapes^48^. Screening of these neoantigens has the potential to predict clinical activity as well as therapeutic resistance.

Tumor tissues from melanoma patients treated with ipilimumab or tremelimumab were used to study the role of somatic mutations as predictive biomarkers for clinical response. Whole genome somatic neoepitope analyses and patient-specific HLA typing were performed in tumors and whole blood samples from 64 patients. It was reported that the neoantigen landscape, as defined through IHC analyses, has a strong association with the treatment response to CTLA-4 blockade^49^. This study strengthens high-throughput IHC analyses using biopsy specimens to clinically validate therapeutic outcomes.

Recent studies revealed that the evolution of the neoantigen profile in NSCLC patients is associated with the response to ICIs. Acquired resistance to immunotherapy was observed in a cohort of 42 patients with NSCLC who were treated with a PD-1 inhibitor alone or in combination with a CTLA-4 inhibitor^48^. The whole genome of paired tissues collected before and after therapy was analyzed for the neoantigen landscape related to therapy resistance. This study reported that loss-of-function mutations coding for neoantigens either by the elimination of tumor clones or by chromosomal truncated gene alteration can result in therapy resistance^48^. Additionally, tumor cells alter the expression of immune suppressive proteins and multiple transcription factors involved in immune functions to acquire resistance against ICIs^50^. Whole-genome analyses performed on tissues obtained from baseline and relapsed tumors of metastatic melanoma patients undergoing pembrolizumab treatment revealed that acquired resistance to ICIs are associated with loss-of-function mutations^51^. Truncated mutations in IFN-receptor-associated JAK1 or JAK2 that cause the loss of IFN-γ function and mutations in the B2M gene, resulting in the loss of MHC-I expression and antigen presentation, are also reported in acquired ICI therapy-resistant samples^51^.

## Genetic signatures

In a retrospective study conducted with a cohort of breast cancer patients with 1- to 5-year tumor relapse versus those with up to 7-year relapse-free survival, Ascierto et al.^52^ screened more than 299 immune-related genes and found that five genes (IGK [IGKC], GBP1, STAT1, IGLL5, and OCLN) were highly overexpressed in patients with relapse-free survival, highlighting their potential as predictive biomarkers. Similarly, RNA expression studies in ipilimumab-treated patients revealed that the number of immune-related genes involved in both innate and adaptive responses were overexpressed in patients with better clinical activity compared with nonresponsive patients. This suggests the importance of a pre-existing immune-active TME for better clinical response to ipilimumab. PD-L1 and PD-L2 copy number alterations (CNA) are also considered potential biomarkers^53^. Budczies et al.^54^ reported PD-L1 CNA in 22 major cancers and found a strong correlation between PD-L1 CNA and mRNA expression levels. The mutation load also correlated with PD-L1 copy number gains.

The mutational loads in exomes also have potential roles as predictive biomarkers for ICIs. Studies have shown that patients with higher mutational loads have greater responsiveness to ICIs^49,55^. Genetic mutations that lead to the expression of immune-related peptides that expand pre-existing T cells or that can be generated in response to immune or other stimuli can increase the efficacy of ICIs^49,56^. JAK3, a member of the Janus kinase signaling pathway, generally found in leukocytes, was reported to have a regulatory role in PD-L1 expression in lymphomas^57^. Mutations that activate JAK3 can cause overexpression of PD-L1 in lymphomas and make them responsive to PD-L1 inhibitors^58,59^.

Mismatch-repair mechanisms are the machinery that protects cells by repairing mutations during DNA replications. A high neoantigen load and high mutational load are associated with an improper mismatch-repair system. The identification of defective mismatch-repair mechanisms may therefore be exploited as potential predictive biomarkers. Mismatch-repair deficiency in pembrolizumab-treated patients with hereditary nonpolyposis colorectal cancer resulted in a high positive response, highlighting the potential of mismatch-repair deficiency as a predictive biomarker^60,61^. Additionally, in a recent study with 53 cancer patients, the objective response rate was 50% in patients with mismatch-repair deficiency, compared to 0% in patients with mismatch-proficient tumors^60^. The mismatch-deficient group, compared with the other group, also showed a longer progression-free survival^61^. Advances in NGS and microarray technologies have made genome-wide screening of potential markers comparatively easier. The accurate prediction of these biomarkers and their use in clinical conditions are suboptimal. However, the development of simple algorithms to read these potential gene signatures from patient DNA is necessary to make these findings clinically applicable. A PanCancer IO 360™ assay was developed by nanoString; the assay profiles TME interactions using a 770 gene panel. This panel evaluates multiple immune processes, including simultaneous assessment of immune evasion in the context of all three immune phenotypes (immune desert, immune excluded and immune inflamed) and supports the prediction of patient responses to a variety of immunotherapies, including ICIs^41^.

## Epigenetic signatures

Epigenetic modifications are complex cellular processes that can modify cellular functions in response to the prevailing environment without altering genetic codes. Multiple epigenetic marks are involved in these complex mechanisms, including DNA methylation, post-transcriptional histone tail modifications, and short noncoding RNAs^62^. Although the association of multiple epigenetic regulatory mechanisms was evaluated in response to immune checkpoint expression and their applicability in combination therapy for synergistic combination, studies on the evaluation of epigenetic modifications as predictive biomarkers are warranted.

The transcriptomic and epigenetic studies on NSCLC show that the hypomethylation of the CTLA-4, PD-1, and PD-L1 promoter regions may be associated with the upregulation of these genes in the TME^63^. It has been shown that in chronic lymphocytic leukemia (CLL), the mRNA and protein levels of PD-1 were elevated and significantly hypomethylated in both promoter and enhancer regions compared to healthy B-cell controls^64^.

miRNAs are small single-stranded RNA sequences that have a critical role in various diseases, including cancer^65^. Reports have shown that five members of the miR-200 family, miR-200a, 200b, 200c, 141, and 429, play pivotal roles in tumor suppression by restricting the epithelial-to-mesenchymal transition (EMT)^66–68^. In human breast cancer cells, it has been reported that expression of PD-L1 decreases with overexpression of miR-200^69^. These reports rationalize the hypothesis that miR-200 might be a promising biomarker for responders treated with anti-PD-L1 antibodies (atezolizumab or durvalumab). A recent study showed that serum miRNA levels correlated with progression-free survival and overall survival in a phase II clinical study on patients with esophageal squamous cell carcinoma (ESCC) treated with nivolumab^70^. Eight miRNAs were found to be associated with a better clinical response, out of which four miRNAs were positively associated with progression-free survival^70^. In contrast, overexpression of miR-34a has been reported as an inducer of CD8^+^ TILs by repressing PD-L1 expression in colorectal carcinoma and NSCLC patients^71,72^. These data suggest that the miRNA-PD-L1 axis might be a promising therapeutic/diagnostic biomarker target in ICI therapy.

## Concluding remarks

Immunological response to ICIs is a complex process. Biomarkers that predict the efficacy of ICI therapy and irAEs should help in patient selection and decision-making by distinguishing between responders and nonresponders. Numerous studies on predictive biomarkers focusing on immune cell infiltration, peripheral blood analyses, PD-L1 overexpression, copy number alterations, neoantigen clonality, mutational landscape, mismatch-repair deficiency, SNPs, transcription factors, and miRNA are currently available (Table 2).

**Table 2:** Predictive biomarkers for progression-free survival and overall survival in patients treated with immune checkpoint inhibitors

Biomarker Category	Nonresponders	Responders
Immune cells	Decreased • Lymphocytes (CD4^+^, CD8^+^)^14^ • B Cells (CD20^+^)^26^ • Activated lymphocytes (CD134^+^, CD137^+^, and FOXP3^+^)^26^ • Natural killer cells (NKp46^+^)^26^	Increased • Peripheral blood absolute lymphocyte count^14,15^ • Absolute eosinophil count^17^ • Relative lymphocyte count^17^ • Tumor-infiltrating lymphocyte (CD4^+^, CD8^+^)^14,74^ • Teff to Treg ratio^27,28^ • Number of activated T cells (CD134^+^, CD137^+^, and FOXP3^+^)^26^ • Monocytes (CD16^+^ and CD68^+^)^26^ • CD8^+^PD-1^hi^CTLA-4^hi^ and CD4^+^FOXP3^-^PD-1^hi^ subpopulations^31,75^ Decreased peripheral blood • Absolute monocyte count^17^ • Myeloid-derived suppressor cells^17^
Protein expression	• Basal level expression of PD-L1^43^ • The loss in IFN-γ signaling in CD8^+^ T cells^47^	Increased • Expression of PD-L1^74^ • PD-L1 copy number gain^53,54^ • Expression of IFN-γ^40,45,47^ • Expression of IDO^47^ • Th1-associated markers^47^ • ICOS pathway^37,65,68^ Decreased LDH level^15–17^
Mutations and neoantigens	• Elimination of neoantigen-expressing tumor clones^48^ • Decreased neoantigen burden^47,76^	• Higher mutational load^49,55^ • Clonal mutations in neoantigens^47,48^ • Mismatch-repair deficiency^60,61^ • Increased neoantigen burden^47^
Gene signatures		• Overexpression of IGK, GBP1, STAT1, IGLL5, and OCLN^52^ • Overexpression of immune-related peptides expanding pre-existing T cells^49,56^ • Activation in JAK3^58,59^
Epigenetic signatures		• Altered methylation pattern of PD-L1^26,63,64^ • Higher serum levels of miRNA^69–72^

Major issues in the development of predictive biomarkers are the dynamic variations in cancer biomarker types and a patient’s genetic makeup. Biopsies obtained from multiple sites of the same patient showed variation in biomarker levels owing to intratumoral heterogeneity. Intense research will develop combination biomarker sets to predict ICI therapy outcomes and avoid irAEs^73^.

Although several predictive biomarker studies are completed and many are underway, the clinical validation of the identified biomarkers is necessary. More integrated approaches should be developed to identify patient-specific choices for checkpoint monotherapies or combination therapies. Moreover, next-generation sequencing techniques should become clinically applicable through the development of simple algorithms to process large quantities of clinical data. In conclusion, biomarker-driven prediction of immune therapy outcomes has the potential to make dramatic changes in cancer immunotherapy.
